# Neurocognitive Outcome and Compensating Possibilities in Children and Adolescents Treated for Acute Lymphoblastic Leukemia With Chemotherapy Only

**DOI:** 10.3389/fpsyg.2019.01027

**Published:** 2019-05-16

**Authors:** Grete Elisabeth Lofstad, Trude Reinfjell, Siri Weider, Trond H. Diseth, Knut Hestad

**Affiliations:** ^1^Department of Psychology, Norwegian University of Science and Technology, Trondheim, Norway; ^2^Department of Child and Adolescent Psychiatry, St. Olav’s University Hospital, Trondheim, Norway; ^3^Division of Paediatric and Adolescent Medicine, Department of Child and Adolescent Mental Health in Hospitals, Oslo University Hospital, Oslo, Norway; ^4^Institute of Clinical Medicine, Faculty of Medicine, University of Oslo, Oslo, Norway; ^5^Department of Research, Innlandet Hospital Trust, Brumunddal, Norway; ^6^Department of Health and Nursing Science, Inland Norway University of Applied Sciences, Elverum, Norway

**Keywords:** acute lymphoblastic leukemia, survivors, neurocognitive, long-term sequelae, compensating possibilities

## Abstract

**Aim:** To examine the neurocognitive outcomes in children and adolescents with acute lymphoblastic leukemia (ALL) in remission who were treated with systemic chemotherapy only (CTO).

**Methods:** Neurocognitive performances in 36 children and adolescents, aged 8.4–15.3 years, in long-term remission from ALL 4.3–12.4 years post diagnosis, without relapse, and with no pre-diagnosis history of neurodevelopmental disorder were compared with 36 healthy controls matched for gender, age, and parents’ socio-economic status. The former patients and the healthy controls completed an extensive battery of standardized neuropsychological tests.

**Results:** Survivors who were treated by CTO obtained significantly lower scores than did healthy controls on the domains of Copy and drawing (*p* = 0.001; Cohen’s *d* 0.85; after controlling for Type 1 errors *q* = 0.006), Arithmetic (*p* = 0.001; Cohen’s *d* 0.80; after controlling for Type 1 errors, *q* = 0.006), and Tactile sensory functions (*p* = 0.008; Cohen’s *d* 0.65; after controlling for Type 1 errors, *q* = 0.03). Fifty percent of the ALL group were more than 1 SD below the control groups mean on Copy and drawing. There was an interaction between age and group (ALL vs. Control, *p* = 0.042) on Copy and drawing, indicating that the youngest ALL patients exhibited the worst performance. The oldest ALL patients performed equal to or better than the controls. A tendency in the same direction was seen for Arithmetic and Tactile sensory functions. The ALL survivors exhibited a steeper rising learning slope on repeated tests, with lower scores on a tactile problem-solving task, tactile sensory tests, verbal memory, and visual attention, but they performed as well as the controls when stimuli were repeated.

**Conclusion:** The results indicate that neurocognitive long-term sequelae in ALL survivors are limited to specific domains – particularly complex drawing, arithmetic, and tactile processing, and novelty processing. Cognitive deficits are shown among the youngest ALL patients. Intervention programs and school programs should account for difficulties with processing new information and taking advantage of repetitions as a strength, which may prevent survivors from falling behind their peers.

## Introduction

Acute lymphoblastic leukemia (ALL) is the most common childhood malignancy, with an incidence of 3.9 per 100,000 children (Gustafsson et al., 1998; Schmiegelow et al., 2010). With the current treatment protocols, the overall 5-year event-free survival (EFS) over the last several decades has approached 90% in the Nordic countries (Schmiegelow et al., 2010). Central nervous system (CNS) prophylaxis to prevent CNS relapse is one of the major improvements in the treatment. ALL is most typically diagnosed in preschool-age children, with a striking incidence peak at age 2–5 years, which is a period of rapid brain maturation and development (Krull et al., 2013).

Acute lymphoblastic leukemia, together with brain tumors, differs from most other childhood cancers in the increased vulnerability for attracting long-term cognitive sequelae. Cranial radiation therapy (CRT), and CRT in combination with chemotherapy (CT) for CNS prophylactic treatment is accepted as a central agent for cognitive sequelae (Campbell et al., 2007). In current treatment protocols, Intensified CT has successfully replaced CRT without reducing the EFS (Schmiegelow et al., 2010). Nevertheless, chemotherapy-only (CTO) protocols usually employ simultaneous administration of different types of drugs, including nucleoside analogs, glucocorticoids, and anti-folates, all of which are suspected to cause both acute and delayed neurotoxicity (Cole and Kamen, 2006).

New research has found reduced brain connectivity in ALL patients treated with CTO, compared to healthy controls, and it is therefore hypothesized that such changes may be associated with the observed cognitive impairments in this group (Kesler et al., 2014). Chemotherapy-based CNS-directed treatment may therefore have a negative impact on neurocognitive functioning in ALL survivors – although to a lesser extent than radiotherapy does (Peterson et al., 2008; Jacola et al., 2016) – and has led to an increased focus on the late effects in long-term survivors of ALL (≥5 years after diagnosis) treated with CTO protocols (Diller, 2011).

We performed a systematic search for studies on ALL and neurocognitive tests in PsycInfo (OVID, 1987–2019) and Medline (OVID, 1946–2019), using subject terms and text words. Subject terms used in Medline were (1) Precursor Cell Lymphoblastic Leukemia-Lymphoma/, (2) Leukemia, Lymphoid/, (3) exp Antineoplastic Agents/, and (4) exp Neuropsychological Tests/. The search strategy was adapted to PsycInfo and supplemented with text word searches. In total, 72 studies were retrieved, with 1 duplicate. Studies published between 1990 and 2019 were included. The full-search strategy can be obtained if needed. We went through the abstracts and deleted 50 articles that did not fit our topic. Following our inspection of the manuscripts, articles featuring cranial radiation therapy and those lacking healthy control groups were also excluded. Among those articles included was one meta-analysis of neurocognitive impairment in childhood ALL, based on 13 articles that met the criterion for inclusion (Peterson et al., 2008). Their conclusion indicated cognitive impairment in processing speed, verbal memory (including working memory), some aspects of executive function (perceptual reasoning skills), and fine motor skills among patients with ALL. The authors state in their discussion, however, that the neuropsychological findings were mixed with some evidence for fine motor, executive function, and verbal memory weakness in ALL survivors.

Turning to the findings from articles not in this meta-analysis, Buizer et al. (2005a) found Visuomotor difficulties restricted to the condition requiring the highest level of control, more in girls than boys, and more in those with a short time since treatment. In another article Buizer et al. (2005b) examined 36 ALL patients (presumably the same ALL patients) with an attentional test, and found that CNS-directed CTO is associated with attentional dysfunction, particularly with intensified treatment protocols. Oliveira-Gomes et al. (2012) concluded that ALL children with CTO displayed mild cognitive deficits that could limit school performance, insertion to jobs, autonomy, and life quality. Their 20 ALL patients exhibited difficulties with executive functions, some problem-solving strategies, self-regulation, cognitive flexibility and inhibitory control, and recall after interference. In a Norwegian study of long-term survivors (Kanellopoulos et al., 2016) (ALL, *n* = 112 control, *n* = 100, mean time 22.6 years, 7–40 years post treatment), ALL patients had normal general intellectual ability but reduced performance in processing speed, executive functions, and working memory compared to peers. Another study (Kunin-Batson et al., 2014) (ALL = 263), examining ALL patients with a mean of 9 years from diagnosis, found mild neurocognitive difficulties, with problems related to verbal cognitive abilities and visual-motor integration. In a recent review, Cheung and Krull (2015) noted that there is limited research on cognitive sequelae after CTO treatment in ALL survivors, and that although most such studies have described cognitive deficits in these patients, the findings have been inconclusive. The effect of CTO on IQ has generally been found to be modest. Lofstad et al. (2009) found a group difference of specific cognitive functions, measured by the Wechsler Intelligence Scale for Children-Third Edition (WISC-III). The deficit in the ALL group was most striking and consistent for the verbal and attention indexes. In addition, lower scores were revealed in complex visual-spatial problem-solving tasks and processing speed for the survivors of early-childhood ALL compared to the matched healthy controls. This is in accordance with findings in a systematic review by Cheung and Krull (2015), who revealed difficulties in attention, processing speed, and executive functions. Others have reported possible late effects in verbal fluency (Espy et al., 2001) and a specific lowering of Verbal IQ (Harila et al., 2009). Longitudinal data from childhood leukemia survivors participating in the Childhood Cancer Survivor Study (CCSS) supported an association between impaired intellectual abilities on the one hand and unemployment and weaker educational gains on the other (Krull et al., 2013). Based on this literature, it is reasonable to conclude, as Peterson et al. (2008) have done, that neuropsychological findings in ALL survivors are mixed.

Changes observed in neurocognitive functions appear to be the result of complex interactions involving genetic predisposition, cancer type, age, and treatment modality (Ross et al., 2004). Younger age at treatment, female gender, intrathecal chemotherapy, and cranial irradiation are all associated with poorer neurocognitive outcomes (Buizer et al., 2009; Kahalley et al., 2013). Importantly, impairment in these domains can impede the ability to learn new information and maintain previously learned information and can lead to declines in neurocognitive functioning (Krull et al., 2008, 2013).

In addition to the observed cognitive impairments seen for ALL patients (Kesler et al., 2014), recent neuroimaging studies indicate that damage to white matter are associated with cognitive deficits in survivors of childhood cancers (Hutchinson et al., 2017).

In sum, during the last decade there has been a growing research focus on neurocognitive sequelae/consequences after treatment for ALL in childhood. We wanted to extend this research by including full neurocognitive assessments typically used in clinical settings.

The aim of the present study is thus to examine neuropsychological differences between survivors of childhood ALL with no relapse, treated with CTO, compared to matched healthy controls, using an extensive neuropsychological test battery generating the possibility of examining if specific areas of cognitive domains are more involved than others. We hypothesized that there would be cognitive deficits in the ALL group compared to the healthy control group. Based on earlier studies, we would expect to find deficits in specific cognitive domains, including verbal and visual-spatial deficits. In addition, we expected difficulties with attention, working memory, processing speed, and executive functions. Thus, the expectation was not for an overall cognitive impairment in these patients, but for their specific inferior cognitive performance compared to healthy controls.

## Materials and Methods

### Participants

The participants in the present study comprised 36 children and adolescents who were long-term survivors of childhood ALL and 36 healthy controls who were matched for age, gender, and socio-demographic status (Table 1). The ALL children and adolescents were recruited from Oslo University Hospital and St. Olav’s University Hospital in Trondheim, Norway. The ALL survivors were treated in accordance with protocols developed in 1992, Nordic Society of Paediatric Haematology Oncology-ALL (NOPHO-1992) used in Denmark, Finland, Iceland, Sweden, and Norway, without CRT.

**Table 1:** Sociodemographic characteristics and treatment variables in 36 children treated for ALL and 36 healthy controls.

	ALL	Healthy
Gender		
Girls: n (%)	19 (52.7)	19 (52.7)
Age at study in years		
Mean (SD)	11.5 (2.0)	11.6 (1.9)
Range	8.4–15.3	8.8–15.1
Family composition; n (%)		
Both parents	27 (75.0)	27 (75.0)
Parent with partner	4 (11.1)	5 (13.9)
Parent single	5 (13.9)	4 (11.1)
Parents: age in years, mean (range)		
Mother	39.6 (30-54)	39.5 (30-52)
Father	43.1 (32-58)	42.6 (30-59)
Parents: education in years, aaa, mean (range)		
Mother	13.8 (10-19)	13.8 (9-19)
Father	14.3 (10-20)	13.6 (9-19)
Economy, self-evaluation; aaa		
Very good; n (%)	3 (8.3)	5 (13.9)
Good; n (%)	21 (58.3)	14 (38.9)
Average; n (%)	9 (25.0)	17 (47.2)
Poor	2 (5.6)	0
Home;		
Own house, n (%)	33 (91.7)	33 (91.7)
Community;		
Urban, n (%)	12 (33.3)	15 (41.7)
Diagnosis and treatment		
Age at diagnosis in years		
Mean (SD)	3.8 (1.5)	
Median	3.6	
Range	1.5–7.5	
Time since diagnosis in years		
Mean (SD)	7.7 (1.9)	
Median	7.9	
Range	4.3–12.4	
Treatment protocols, n (%)		
Standard risk	14 (38.9)	
Intermediate risk	16 (44.4)	
High risk 1	4 (11.1)	
High risk 2	2 (5.6)	

*No significant differences were seen between the groups. Missing data in the ALL group are indicated by aaa; *n* = 35. We were missing data on one of the parents who did not respond*.

The treatments based on the NOPHO-ALL-1992 protocol were initiated from May 1992 through 1999, with patients grouped as standard-risk, intermediate-risk, high-risk aged under 5 years, high-risk 2 aged over 5 years, and very high-risk. These treatment protocols included pulses of high-dose methotrexate (5–8 g/m^2^) isolated or in combination with high-dose cytosine arabinoside (total dose 12 g/m^2^) plus multiple intrathecal injections of methotrexate as a CNS-targeted treatment, prednisolone, doxorubicin, vincristine, L-asparaginase, and oral 6-mercaptopurine (Gustafsson et al., 1998). Patients were included if they met the following inclusion criteria: 4 years or more post-diagnosis; in continuous remission since the initial treatment, with no relapses; and completion of a single course of treatment without CRT. A total of 51 children and adolescents from the patient pools provided by the two hospitals met the inclusion criteria and were contacted by regular post. Thirty-six children (70.6%), 19 girls and 17 boys, and their parents agreed to participate in the study. The group had a mean age of 11.5 years (range 8.4–15.3 years) and was 4.3–12.4 years post diagnosis (mean = 7.7). The 15 children who did not participate (12 girls and 3 boys) reported difficulties in finding time for the nearly 2-day-long evaluation period as the main reason for not participating. Other parents declined because they did not want to remind their children of their cancer treatment. Norway is a small country, with a total of five University Hospitals, two of which – Oslo University Hospital in Oslo and St. Olav’s University Hospital in Trondheim – took part in this study. These two university hospitals diagnosed approximately 77 of 146 children with ALL in Norway during this time span (1989–1995). This figure amounts to 52.7% of the total sample in Norway. There were 14 children belonging to the standard risk group, 16 to the intermediate group, and 6 to the different high-risk groups. To maximize the homogeneity in patient group and exclude confounding factors, participants in the very high-risk group treated with cranial radiation therapy or bone-marrow transplantation and/or participants with possible central nervous system involvement or B-cell leukemia or other neurodevelopmental syndrome or diseases were excluded from the study.

The physically healthy children and adolescents in the comparison group were recruited from four public schools and matched by gender, age, and parents’ socioeconomic status. The schools were selected to match demographics. As in the patient group, only pupils from Nordic families with a Nordic first language and without any known neurodevelopmental syndrome or disorder were recruited. The participation rate, among the controls was 79%, with a mean age of 11.6 years (range 8.8–15.1 years). There was no gender difference in participation or refusal to participate in the study. Additional descriptions of the participants have been published elsewhere (Lofstad et al., 2009).

### Methods

Neuropsychological functions were assessed with an extensive battery of neuropsychological tests representing a wide range of neuropsychological functions:

A. An extended Halstead-Reitan neuropsychological test-battery (Reitan and Wolfson, 1993), including (1), Aphasia screening test, (2), Category Test, (3), Tactual Performance Test, (4), Trail Making Test A and B, and (5), Seashore Rhythm Test;
B. Matthew-Kløve Motor Steadiness Battery and Reitan-Kløve Sensory Perceptual Examination (Reitan and Davison, 1974), including (1), Maze Coordination, (2), Finger and Foot Tapping, (3), Dynamometer (handgrip strength), (4), Grooved Pegboard, (5), Tactile Form Recognition (stereognosis), (6), Finger Agnosia, and (7), Fingertip Writing;
C. Developmental Drawing (Yeates, 1994), including a paper-and-pencil design-copying test;
D. Knox Cube Test (Bornstein, 1983);
E. Boston Naming Test (Baron, 2004);
F. Controlled Oral Word Association Test (COWAT FAS) (Benton and Hamsher, 1976);
G. Children Auditory Learning Test-2 (CAVLT-2) (Talley, 1993);
H. Rey-Osterrieth Complex Figure Test (RCFT) (Meyers and Meyers, 1995);
I. Stroop Color-word Test (Golden, 1978);
J. Paced Auditory Serial Addition Test (PASAT) (Gronwall, 1977); and
K. Conners’ Continuous Performance Test (CPT) (Connors, 1995).

In addition, the WISC-III was used. We have previously reported from WISC-III (Lofstad et al., 2009), but found it useful to include this measure with the other tests used in the assessment. In the previous publication, these results were not converted to *T*-scores based on the healthy controls or included in domain summary *T*-scores.

The parents were asked to complete a standardized questionnaire regarding case history and demographic data. The WISC-III was presented according to test protocol, beginning with the Information subtest ending with the Mazes. The typical neuropsychological tests were presented in a random order to ensure that test tiredness, boredom, or other such factors would not influence the results.

### Procedure

Written permission to contact the parents of children treated for ALL in their patient pools was provided by the Paediatric Clinics at Rikshospitalet and St. Olav’s Hospital. Written information about the project; parent and patient (adolescent) consensus forms; and self-addressed, stamped envelopes were sent by regular post to parents of the children who met the inclusion criteria (*n* = 51). If forms were not returned within 3 weeks, the parents were contacted by phone. All children whose parents had provided informed consent were examined in a quiet room at the Division of Paediatric and Adolescent Medicine at the Oslo University Hospital, Oslo, or at the Neuropsychological Clinic at the Norwegian University of Science and Technology, Trondheim. The WISC-III was administered on the first day, together with interviews with the patient and parents. All neuropsychological tests were presented on the second day in two sessions, with a 1-hour break between sessions.

The first author, an experienced neuropsychologist (G.E.L), administered the tests. Additional descriptions of the procedure have been published elsewhere (Lofstad et al., 2009). To assemble the control group, the county borough councils for schools in one urban and one rural county were contacted to discuss the demographics of different schools and to obtain permission to contact school headmasters in their respective counties. Two Trondheim city headmasters (one from an elementary school and one from a junior high school) were contacted and asked to create a sample of two girls and two boys in each group, by drawing lots from gender and age-specific (according to school year) pupil lists. The participants from these two schools and the children from the ALL group were divided by gender, ranged by age in months, and matched by age. The age in months for ALL survivors without healthy matches was then recorded, after which headmasters from the rural county of Nord-Trøndelag were asked to select healthy matches by gender and age in months. Headmasters sent written information and consent forms to selected families, who were subsequently contacted by phone. Headmasters were instructed not to include pupils with known neurodevelopmental diseases diagnosed by the specialist health services. Once parents and adolescents gave their informed consent, the neurocognitive test batteries were administered in a quiet room at their respective schools.

The Regional Committee for Medical and Health Research Ethics – Central Norway (092-02) – approved the study. All participants and their parents provided written informed consent, and the research was completed in accordance with the Declaration of Helsinki.

### Statistics

The match of the two groups was evaluated by comparing the demographical variables using Independent Sample *t*-tests or Pearson χ^2^. The results on each neuropsychological subtest were converted to *T*-scores, based on the test results from the matched control group, thereby providing results on the same scale, which would make it easier to see differences in performance between the two groups on the different tests. The different test scores were divided into separate domains, and summary *T*-scores was calculated for each domain with a Mean of 50 and a SD of 10 for the control group. The neuropsychological outcome for the ALL patients was compared to those of the healthy controls, using Independent Sample *t*-test. There were some missing data in our material in the ALL population. One WISC-III test protocol was accidentally destroyed before it was entered into the database. Furthermore, there were missing values on three participants on the Connors continuous performance test (due to technical difficulties), three on the PASAT test (tiredness and hesitation regarding mathematical tests), two on the Stroop test, and one on Developmental drawing. On these subjects, a mean score for the group was inserted. Because participants were closely matched, we followed the same procedure for control participants. On the missing WISC-III subtests, for instance, the results for matched controls were substituted with the groups mean score. Because all comparisons between the two groups could result in Type 1 errors, a false discovery rate (FDR) procedure was performed in order to control for multiple comparisons (Benjamini and Hochberg, 1995) on the domain summary *T*-scores. The values from the FDR are marked as *q*-values.

We also examined for differences between the ALL groups related to treatment protocol (i.e., standard risk, intermediate risk, and high risk).

The data were analyzed using the Statistical Package for Social Sciences (SPSS, version 24.0, SPSS Inc., Chicago, IL, United States). The statistical program R was used for the FDR^1^.

## Results

The demographical variables revealed no significant differences between patients and healthy controls (see Table 1).

### Neuropsychological Status

As shown in Table 2, the ALL survivors scored significantly lower than the healthy controls on the summary scores of the following domains: Tactile and sensory function (*p* = 0.008), Abstraction and set-shifting (*p* = 0.045), Processing speed (*p* = 0.036), Copy and drawing (*p* = 0.001), and Arithmetic (*p* = 0.001). After controlling for multiple comparisons differences in summary scores between the two groups, however, ALL survivor scores were less than those of healthy controls only for Copy and drawing, Arithmetic, and Tactile and sensory functions (see Table 2).

**Table 2:** Independent sample *t*-test on neuropsychological test results in 36 ALL survivors compared to 36 matched (matched for age education and socio-economic parent status) healthy controls.

Function	Patients treated for acute lymphoblastic leukemia Mean (SD) *T*-scores	Matched healthy controls Mean (SD) *T*-scores	Confidence interval and *p*-value, Cohen’s *d*; false discovery rate, *q*-value for summary scores
**Motor tests**			
Finger tapping, dominant hand	48.9 (9.9)	50.0 (10.0)	-5.8 to 3.5; *p* = 0.63
Finger tapping, non-dominant hand	47.6 (9.8)	50.0 (10.0)	-7.1 to 2.2; *p* = 0.30
Foot tapping, dominant hand	50.8 (12.2)	50.0 (10.0)	-4.5 to 6.0; *p* = 0.77
Foot tapping, non-dominant hand	47.0 (10.9)	50.0 (10.0)	-7.9 to 1.9; *p* = 0.22
Grooved Pegboard dominant hand	48.3 (14.3)	50.0 (10.0)	-7.5 to 6.1; *p* = 0.57
Grooved Pegboard non-dominant hand	51.5 (10.6)	50.0 (10.0)	-3.3 to 6.3; *p* = 0.54
Kløve-Matthew, motor steadiness:			
Steadiness Maze Counter dominant hand	44.1 (19.1)	50.0 (10.0)	-13.0 to 1.3; *p* = 0.10
Steadiness Maze Counter non-dominant hand	51.2 (12.7)	50.0 (10.0)	-4.2 to 6.6; *p* = 0.65
Steadiness Maze Timer dominant hand	44.6 (21.6)	50.0 (10.0)	-13.3 to 2.5; *p* = 0.18
Steadiness Maze Timer non-dominant hand	49.0 (13.5)	50.0 (10.0)	-6.6 to 4.6; *p* = 0.72
Grip Strength dominant hand	50.2 (14.9)	50.0 (10.0)	-5.8 to 6.1; *p* = 0.95
Grip Strength non-dominant hand	49.9 (15.4)	50.0 (10.0)	-6.2 to 6.0; *p* = 0.97
**Summary score motor tests**	**48.1 (14.3)**	**50.0 (10.0)**	-**7.7 to 3.9; *p* = 0.51**
			**Cohen’s *d* 0.15; *q* = 0.51**
**Tactile sensory**			
Error in Tactile Form Recognition dominant hand	41.8 (22.6)	50.1 (10.0)	-16.5 to -0.1; *p* = 0.045
Error in Tactile Form Recognition non-dominant hand	45.9 (14.3)	49.9 (10.0)	-9.8 to 1.8; *p* = 0.18
Finger-tip Number Write dominant hand	49.2 (10.1)	50.0 (10.0)	-5.5 to 3.9; *p* = 0.74
Finger-tip Number Write non-dominant hand	44.8 (11.1)	50.0 (10.0)	-10.1 to -0.22; *p* = 0.041
**Summary Score Tactile sensory**	**41.0 (16.8)**	**50.0 (10.0)**	-**15.5 to**-**2.4; *p* = 0.008**
			**Cohen’s *d* 0.65; *q* = 0.03**
**Abstraction and set shifting**			
Halstead-Reitan Test Battery, (HRB), Category test	48.2 (10.3)	50.0 (10.0)	-6.6 to 3.0; *p* = 0.46
WISC III, similarities	39.6 (11.6)	50.0 (09.9)	-15.5 to -5.4; *p* < 0.001
Trail Making Test part B	50.3 (13.9)	50.0 (10.0)	-5.4 to 6.0; *p* = 0.91
**Summary Score Abstraction and set shifting**	**44.6 (12.7)**	**50.0 (10.0)**	-**10.8 to**-**0.01; *p* = 0.05**
			**Cohen’s *d* 0.47; *q* = 0.1**
**Processing speed**
Trail Making Test part A	49.0 (12.1)	50.0 (10.0)	-6.3 to 4.2; *p* = 0.69
STROOP test, interference	47.3 (10.2)	50.0 (09.9)	-7.4 to 2.0; *p* = 0.26
Tactile Form Recognition dominant hand	42.3 (15.6)	50.0 (10.0)	-13.9 to -1.5; *p* = 0.015
Tactile Form Recognition non-dominant hand	45.8 (11.9)	50.0 (10.0)	-9.3 to 1.0; *p* = 0.11
WISC III, Digit Symbol	45.8 (12.2)	50.0 (10.0)	-9.5 to 1.0; *p* = 0.11
WISC III, Symbol search	46.6 (11.5)	50.0 (10.0)	-8.5 to 1.7; *p* = 0.18
**Summary Score Processing speed**	**44.0 (12.9)**	**50.0 (10.0)**	-**11.5 to**-**0.6; *p* = 0.030**
			**Cohen’s *d* 0.53; *q* = 0.08**
**Copy and drawing**			
Rey Complex Figure Test, copy	35.9 (23.3)	50.0 (10.0)	-22.5 to -5.7; *p* = 0.002
Developmental Drawing	40.8 (14.8)	50.0 (09.9)	-15.1 to -3.4; *p* = 0.003
**Summary Score Copy and drawing**	**35.4 (22.1)**	**50.0 (10.0)**	-**22.7 to**-**6.5; *p* = 0.001**
			**Cohen’s *d* 0.85; *q* = 0.006**
**Spatial tests**			
Tactual performance test dominant hand	43.2 (16.0)	50.0 (10.0)	-13.1 to -0.6; *p* = 0.03
Tactual performance test non-dominant hand	48.5 (13.0)	50.0 (10.0)	-7.0 to 3.9; *p* = 0.57
Tactual performance test both hands	49.8 (11.4)	50.0 (10.0)	-5.3 to 4.9; *p* = 0.93
WISC III Picture Completion	50.0 (12.4)	50.0 (10.0)	-5.3 to 5.3; *p* = 1.0
WISC III Picture arrangement	45.6 (14.6)	50.0 (10.0)	-10.3 to 1.5; *p* = 0.14
WISC Block design	41.7 (13.2)	50.0 (10.0)	-13.8 to -2.3; *p* = 0.004
WISC III Object Assembly	40.7 (24.8)	50.0 (10.0)	-18.2 to -0.41; *p* = 0.04
WISC III Maze	50.1 (11.7)	50.0 (10.0)	-5.0 to 5.2; *p* = 0.96
**Summary Score Spatial tests**	**44.2 (15.8)**	**50.0 (10.0)**	-**12.0 to 0.5; *p* = 0.07**
			**Cohen’s *d* 0.44; *q* = 0.1**
**Verbal memory**			
Child Auditory Verbal Learning Test-2 (CAVLT), immediate recall	47.9 (8.9)	50.0 (10.0)	-6.6 to 2.3; *p* = 0.34
CAVLT, delayed recall	47.3 (14.0)	50.0 (10.0)	-8.4 to 3.1; *p* = 0.36
**Summary Score Verbal memory**	**47.1 (12.2)**	**50.0 (10.0)**	-**8.2 to 2.3; *p* = 0.26**
			**Cohen’s *d* 0.27; *q* = 0.29**
**Visual memory**			
RCFT immediate recall	42.9 (15.9)	50.0 (10.0)	-13.4 to -0.9; *p* = 0.027
RCFT delayed recall	42.5 (16.1)	50.0 (10.0)	-13.8 to -1.2; *p* = 0.021
HRB TPT-memory	54.3 (9.2)	50.0 (10.0)	-0.23 to 8.8; *p* = 0.06
HRB TPT-localization	50.2 (10.8)	50.0 (10.0)	-4.7 to 5.1; *p* = 0.94
**Summary score Visual memory**	**46.6 (14.5)**	**50.0 (10.0)**	-**9.2 to 2.5; *p* = 0.26**
			**Cohen’s *d* 0.27; *q* = 0.29**
**Verbal tests**			
Letter fluency, COWAT FAS	53.1 (12.7)	50.0 (10.0)	-2.3 to 8.4; *p* = 0.26
Boston Naming Test	48.9 (13.0)	50.0 (10.0)	-6.6 to 4.4; *p* = 0.69
WISC III Information	46.6 (13.0)	50.0 (10.0)	-8.8 to 2.2; *p* = 0.22
WISC III Vocabulary	41.8 (15.1)	50.0 (10.0)	-14.2 to -2.0; *p* = 0.008
WISC III Reasoning	40.0 (13.1)	50.0 (10.0)	-15.4 to -4.3; *p* = 0.001
**Summary score Verbal tests**	**45.1 (14.7)**	**50.0 (10.0)**	**-10.8 to 1.1; *p* = 0.10**
			**Cohen’s *d* 0.39; *q* = 0.16**
**Arithmetic**			
Paced Serial Addition Test (2 × 20 item)	42.8 (16.5)	50.0 (10.0)	-13.8 to -0.18; *p* = 0.03
Mental Calculation (3 × 17)	43.4 (12.7)	50.0 (10.0)	-1.0 to -0.10; *p* = 0.02
WISC III Arithmetic	42.1 (9.5)	50.0 (10.0)	-12.4 to -3.1; *p* = 0.001
**Summary Score Arithmetic**	**41.2 (12.0)**	**50.0 (10.0)**	**-14.0 to -3.6; *p* = 0.001**
			**Cohen’s *d* 0.80; *q* = 0.006**
**Attention**			
Knox cube Test	45.8 (12.7)	50.0 (10.0)	-9.6 to 1.2; *p* = 0.13
Seashore Rhythm	49.6 (11.7)	50.0 (10.0)	-5.5 to 4.7; *p* = 0.88
WISC III Digit Span	46.1 (8.9)	50.0 (10.0)	-8.3 to 0.69; *p* = 0.0.09
Connors Continuous Performance Test (CPT),
Over-all Index	46.0 (10.0)	50.0 (10.0)	-8.8 to 1.1; *p* = 0.09
STROOP color	51.0 (10.9)	50.0 (10.0)	-4.1 to 6.1; *p* = 0.68
STROOP word	47.0 (12.9)	50.0 (10.0)	-8.6 to 2.6; *p* = 0.27
**Summary Score Attention**	**46.0 (12.2)**	**50.0 (10.0)**	-**9.2 to 1.3; *p* = 0.14**
			**Cohen’s *d* 0.35; *q* = 0.19**

*The formula for computing *T*-score were: (((Raw score – Mean of control group)/SD of control group) ^∗^ 10) + 50, when a high raw score is a good performance. When a high raw score represents a poor performance the formula is: (-((Raw score – Mean of control group)/SD of control group) ∗ 10) + 50. For the summary scores, every *T*-score in the domain were used in the calculation of domain *T*-scores. Cohen’s *d* effect size: 0.20 considered small, 0.50 considered moderate, 0.80 considered large. False discovery rate is calculated based on the 11 summary *T*-scores, giving a *q*-value* Raw scores converted to *T*-scores based on the healthy controls.

When we examined Copy and drawing in greater detail to establish how many that might have a cognitive deficit, 18 (50.0%) of the 36 were more than 1 SD below the mean summary score of 50 (5 of the controls, 13.9%) (χ^2^ = 0.001). Regarding the Tactile sensory summary score, 16 (44.4%) of 36 ALL participants were more than 1 SD below the control groups’ mean score of 50. Five (13.9%) of the controls had such a low score (χ^2^ = 0.004). On the Arithmetic sensory summary score, 16 (44.4%) of the ALL patients and 7 (19.4%) of the controls were more than 1 SD below the mean of 50 (χ^2^ of 0.02). With a more conservative measurement regarding how many that might have a cognitive deficit, the level of significant differences between the two groups were the same. Sixteen (44.4%) of the 36 were more than 1 ½ SD below the mean summary score of 50 (3 of the controls, 8.3%) (χ^2^ = 0.001) on Copy and drawing. On the Tactile sensory summary score, 12 (33.3%) out of 36 ALL participants were more than 1 ½ SD below the control groups’ mean. Three the controls had such a low score (χ^2^ = 0.009). On the Arithmetic sensory summary score, 12 (33.3%) of the ALL patients and 4 (11.1%) of the controls were more than 1 ½ SD below the mean of 50 (χ^2^ of 0.02).

There were no significant differences between the groups on the summary *T*-scores for the following domains: Motor function, Spatial function, Verbal memory, Visual memory, Verbal tests, and Attention. Individual subtests within these domains differed between the groups, however, as can be seen on Table 2.

Analyses of the performance/learning process and improvement over trials in the two groups revealed that the ALL group had a tendency to inferior scores compared to the control group on the first presentation, but not at the second or third trial. On TPT right hand, for instance, the ALL group had a mean raw score on 6.72 min (SD; 3.5) vs. 5.21 (SD; 2.2) for the controls (*T*-score 43.2 vs. 50.0; *p* = 0.034). On left hand, the raw score were 4.73 (SD; 1.4) vs. 4.40 min (SD; 1.2), (*T*-score 48.4 vs. 50.0, *p* = 0.57), and both hands 2.29 min (SD; 1.4) vs. 2.26 (SD; 1.2) (*T*-score 49.8 vs. 50.0, *p* = 0.93). The same tendency can also be seen in Table 2 for the Tactile Form recognition test.

The Knox cube test was presented twice. In the first presentation, the difference between the two groups was close to significant (*p* = 0.058), with raw scores of 12.36 for the ALL patients vs. 13.33 for the controls (*T*-score 44.8 [SD; 12.8] vs. 50.0 [SD; 10.0]). The second trial yielded almost identical results – 13.31 vs. 13.37 – which is far from significant (*T*-score 48.1 [SD; 11.5] vs. 50.0 [SD; 10.0], *p* = 0.46]). On CAVLT-2, the ALL patients had a close to significant result on the first recall (*p* = 0.072) but not on the other immediate recalls or the delayed recall, in which the scores where more similar (*T*-score ALL first, second, third, fourth and fifth trial [SD]: 45.49 [11.0], 48.87 [11.5], 52.79 [9.1], 50.94 (12.3), 49.27 [14.1]). For the interference list and the delayed recall, the *T*-score of the ALL patients *T*-scores were 49.4 and 47.3. Neither score was significantly different from the control groups’ scores, although there was a small tendency for some reduction of the delayed score.

When comparing the different treatment protocols in the ALL group relative to Copy and drawing, no differences could be observed (data not shown). Nor were there observable differences in the Arithmetic or Tactile sensory summary scores.

**Figure 1:**
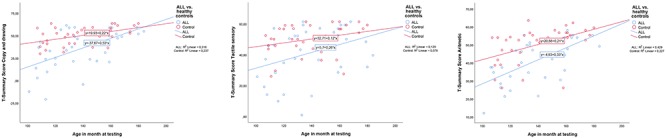
Interactions between age and group (acute lymphoblastic leukemia or controls). From left: Copy and drawing; Arithmetic; Tactile sensory. Age is defined in months. ALL, acute lymphoblastic leukemia. As can be seen from the figures, only Copy and drawing showed a significant interaction (*F* = 4.294, *p* = 0.042). However, the tendency went in the same direction for Arithmetic, and Sensory tactile *T*-scores to close the gap in neuropsychological performance between ALL patients and healthy controls with increasing age.

Gender and age (defined in months) interactions with belonging to (ALL or Control) group were also tested. No interaction was observed with gender, but there was an interaction for age between group for Copy and drawing, with the largest differences appearing in the youngest age group (interaction treatment group and age *F* = 4.294, *p* = 0.042). Among the oldest participants, there are no difference in performance between ALL and control participants. Neither were there interactions between age and gender with group for Sensory tactile and Arithmetic area summary scores, but the findings went in the same direction as for Copy and drawing (see Figure 1).

We also examined if there was a correlation between age and time since diagnosis. A positive correlation was found: More time had passed since the diagnosis for the oldest participants (Pearson Correlation, *r* = 0.7).

## Discussion

In the present study, ALL survivors performed below the healthy controls in 3 of the 11 summary scores examined, after controlling for multiple comparisons. These domains were: Copy and drawing, Arithmetic, and Tactile sensory tests. This finding supports the hypothesis that early childhood ALL treated with CTO influences the subsequent CNS development and is followed by neuropsychological performance inferior to their peers in specific neuropsychological domains. In other cognitive domains specified in the hypothesis no differences were found.

For Copy and drawing, the Cohen’s *d* showed an effect size of 0.85, which is considered large. For the two other domains, Arithmetic and Tactile sensory effect sizes were 0.80 and 0.65, respectively, which are considered as high and moderate effect sizes. The mean level of difference between the two groups was more than 1 SD for Copy and drawing and a much higher SD (17.7 vs. 8.0) in the ALL group than in the control group. For the two other domains, the mean differences ware less than one 1 SD, but the variation/dispersion was larger in the ALL group. Also, there were significant more participants in the ALL groups compared to controls who were more than 1 or 1 ½ SD below the mean summary *T*-score of 50 on Copy and drawing, Tactile sensory and Arithmetic summary scores. With such values, we consider the cognitive difficulties to be clinically significant for the ALL group. The ALL patients demonstrated specific difficulties, which cannot necessarily be detected without carefully neuropsychological testing.

These findings support previous findings showing that early childhood ALL treated with CTO influences subsequent CNS development and is followed by inferior neuropsychological performance compared to their peers in specific neuropsychological domains (Liu et al., 2018). Specifically, our results revealed that the ALL group showed difficulties in tests measuring Copy and drawing, Arithmetic difficulties, and difficulties with Tactile sensory functions.

Difficulties in copying and drawing/visual-spatial constructional ability, as measured by Beery’s Developmental Test of Visual-Motor Integration (VMI), have been documented in other studies (Precourt et al., 2002; Kaemingk et al., 2004). In the present study, however, the survivors scored at same level as the controls on motor function (speed and steadiness), which strengthens the likelihood that this finding is caused by a difficulties in visual-spatial function, which may also be related to mathematical thinking. Previous studies have documented a decline in visual-spatial abilities (Performance IQ) (Montour-Proulx et al., 2005; Jansen et al., 2006) and processing speed (Peterson et al., 2008).

Reports of stereognostic ability and speed in tactile perception in ALL survivors treated with CTO has to our knowledge not been documented before.

The variation/dispersion, taken together with the mean level of performance indicate that not all ALL patients experience cognitive difficulties. Fifty percent or more were within 1 SD of the mean of 50 on the three summary scores with the largest differences between the test groups. In light of the interaction analysis that we performed, it was clearly seen that it was the youngest children with the shortest time since diagnosis that showed the largest cognitive difficulties. We therefore suggest the relatively optimistic view that with time and aging the cognitive difficulties will be reduced. However, our study do not have enough data to address this issue with certainty. It could also be that there is some type of selection bias occurring since only 70.6% of invited participants consented and took part in the study. There should be attention given to the fact that many studies have documented that neurocognitive functioning has declined over time after radiation-free CTO treatment of childhood ALL. The sample in the present study was still in the childhood and adolescence, and may not have grown into the typical problems regarding attention and executive function yet, as found by previous studies focusing on longer-term survivors (Krull et al., 2013; Cheung and Krull, 2015; Kanellopoulos et al., 2016). Our finding that the older participants demonstrated good cognitive performance, however, may work against the possibility that, with increasing demands and task complexity, problems regarding attention and executive function may arise. Liu et al. (2018) found executive function problems to be more prevalent than attention problems in a long-term follow-up situation, and they emphasized that survivors should be monitored for neurocognitive problems well into long-term survivorship, regardless of whether they showed attention problems at the end of therapy.

Furthermore, the survivors and controls scored at a comparable level on measures of verbal fluency, the ability to name known objects, and motor function (both speed and steadiness). Thus, their tendencies for lower scores on CAVLT-2 first recall and the first tactile problem solving are unlikely to be caused by a verbal or motor function deficit.

On learning tasks, the ALL group appears to start below the controls, catch up on repeated learning trials and showing a more steeply rising learning slope than did the healthy controls on the second trial the ALL patients are equal to, or, more precisely, are not different from the control group. Thus, the ALL group seems to benefit from task repetition. This was seen on the Tactual performance test and the Tactile sensory tests, with significant differences on the first trial. On the Knox cube test, there was a borderline significant result at the first performance (*p* = 0.058), with closer to equal test results on the second trial. On the California Verbal Learning test over 5 trials, the difference at the first trial was close to significant (*p* = 0.072). Improvement came with the second trial, and the ALL patients performed equal to or better than (but not significantly different from) the control group over the next trials. The tendency is small but systematic.

These findings could indicate that the ability of ALL survivors to process and encode new information and handle complex multifunctional tasks is impaired. The ability to process novel stimuli is also a central contributor in the performance of working memory tests. The steeper rising learning slope, however, may also indicate an important compensatory mechanism. Our findings that ALL survivors have a tendency toward difficulties in processing novel stimuli are in accordance with the findings of Kaemingk et al. (2004), who observed lower scores on tests of Story Memory, and those of Precourt et al. (2002), who reported a divergent wordlist learning slope among ALL survivor girls compared to healthy controls. In the latter study, the survivors who were treated with CTO reached the same level of performance as controls only at the fourth repetition, without a decrease in the slope. Furthermore, Hill et al. (1997) reported deficits in visual-spatial and verbal single-trial memory tasks: After multiple trials, the survivors reached the same level as the controls on verbal tasks but not on the visual-spatial task, which may indicate deficits in learning for spatial functioning or more complex stimuli, even after repetition.

Contrary to the common perception that cancer is related to increased fatigue, the clinical impression was that the ALL survivors were comparable to those of the healthy controls on the attention domain, which includes a measure of sustained attention, and also that the ALL survivors benefited from repetition. It is interesting to note that the ALL survivors performed at comparable levels to the controls on the selected tests of attention. The finding of intact attention is somewhat surprising and contradicts the findings of Cheung and Krull (2015), who documented difficulties in this domain in their systematic review.

### Strengths and Limitations

Several strengths of this study are the comprehensive neuropsychological evaluation used; the inclusion of 70.6% of the cohort treated for childhood ALL in the two hospitals following the inclusion criteria; the close match between ALL survivors and the healthy control group for gender, age, and socio-economic variables; the within-group homogeneity of the groups; and the fact that all participants were tested by the same experienced clinical neuropsychologist.

The findings of this study must be interpreted with some caution, however, because the sample is relatively limited. Furthermore, most neuropsychological tests are not pure measures of only one specific domain. Consequently, most of the neuropsychological tests that were included measure aspects of several domains. For instance the Arithmetic test from WISC-III was placed within the domain of arithmetic in this study; but it clearly also measures aspects of working memory and attention. Thus a low score could result from working memory or attention problems rather than difficulties related to arithmetic (and vice versa).

Furthermore, several of the tests on which the ALL survivors performed poorly (WISC-III Arithmetic and Digit span and PASAT) could also be labeled measures of working memory. Moreover, the ALL survivors were tested at Oslo University Hospital Rikshospitalet or at the Neuropsychological Clinic in Trondheim, whereas the healthy children were tested at their primary school or at the university clinic, a difference that made it impossible for the test leader to be blinded to the subject’s group affiliation. Altogether, these factors may limit our ability to document smaller impairments and make generalizations from our results.

## Conclusion

The data in this study indicate that long-term survivors of early childhood ALL treated with CTO suffer from neuropsychological sequelae in copying and drawing, tactile sensory function, and arithmetic. This study also documented a steeper learning slope in tests of repeated trials among the ALL survivors than among the controls. The rote memory of ALL group members for repeated stimuli reached the same level or even slightly better than that of the controls after repetitions, however. Thus the changes in the learning curve documented in this study must be examined further. There was an interaction between groups (ALL vs. Control) and age, indicating that there is a change toward better (i.e., good) cognitive performance in the oldest ALL group.

Intervention and school programs must account for the deficits seen in this study. The survivors’ ability for sustained attention and the ability to gain from repetition may serve as an important strength and compensatory mechanism, and may prevent survivors from falling behind their peers.

## Ethics Statement

The study was approved by the Regional Ethics Committee for Medical Research (Helse-Midt 092-02). All participants and their parents provided written informed consent, and the research was completed in accordance with the Declaration of Helsinki.

## Author Contributions

GL and TR designed the study. GL wrote the first draft of the manuscript. SW contributed especially to the methods regarding neuropsychology. TD and KH supervised the study from beginning to end. TD contributed especially with competence related to cancer. KH did the neuropsychological analysis and supervised the neuropsychological performance. All authors participated in the writing process.

## Conflict of Interest Statement

The authors declare that the research was conducted in the absence of any commercial or financial relationships that could be construed as a potential conflict of interest.

## Footnotes

1 https://www.r-project.org/
